# Right anterior section graft for living-donor liver transplantation: A case report

**DOI:** 10.1097/MD.0000000000015212

**Published:** 2019-05-13

**Authors:** Jonathan Geograpo Navarro, Gi Hong Choi, Myoung Soo Kim, Yoon Bin Jung, Jae Geun Lee

**Affiliations:** Department of Surgery, Yonsei University College of Medicine, Seoul, Korea.

**Keywords:** anterior section graft, case report, liver cirrhosis, living-donor liver transplantation

## Abstract

**Rationale:**

In living-donor liver transplantation (LDLT), the right lobe graft is commonly utilized to prevent small-for-size syndrome, despite the considerable donor morbidity. Conversely, the feasibility of the left lobe graft and the right posterior section graft in smaller-sized recipients is now commonly employed with comparable outcomes to right lobe grafts. The efficacy of the right anterior section graft has rarely been reported.

**Patient concerns:**

A 56-year-old man, a heavy alcoholic beverage drinker for 20 years, presented in the emergency department with massive ascites and lethargy. He was previously admitted twice due to bleeding esophageal varices.

**Diagnosis:**

He was diagnosed with hepatic encephalopathy coma due to alcoholic liver cirrhosis. The Child–Turcotte–Pugh score was 11 (class C), and the Model for End-stage Liver Disease score was 21.62.

**Intervention:**

A LDTL was offered to the patient as the best treatment option available. The patient's 26-year-old son was found to be the only donor-compatible candidate for the LDTL.

Preoperatively, the right lobe of the donor occupied 76.2% of the total liver volume exposing the donor to a small residual liver volume. The right posterior section and left lobe volumes were insufficient, providing a graft-to-recipient weight ratio of 0.42% and 0.38%, respectively. However, the right anterior section could fulfill an acceptable GRWR of 0.83%. Thus, a living donor right anterior sectionectomy was performed.

**Outcomes:**

Clinical signs and symptoms and liver function improved following anterior section graft transplantation without complications.

**Lesson:**

The procurement of anterior section graft is technically feasible in selected patients, especially in high-volume liver centers.

## 1 Introduction

Since its introduction in 1994,^[1]^ living-donor liver transplantation (LDTL) has been considered the current standard of treatment for both hepatocellular carcinoma^[2]^ and for patients with the end-stage liver disease.^[3]^ Over the past decades, the revolution of surgical techniques and growing experience in liver transplantation have resulted in good and promising long-term survival.^[4]^ Although it has been widely accepted, LDTL continues to be a challenge due to its intrinsic risks. First, the healthy donor is subject to the inherent risk of the procedure itself,^[5]^ as well as the risk of having a small remnant liver volume after the surgery. Second, there is a potential for the occurrence of small-for-size graft syndrome (SFSS) in the recipient.^[6]^ Thus, to ensure the donor's safety and an excellent outcome in the recipient, proper selection and determination of the graft size are of paramount importance.

In the adult-to-adult LDTL, the use of the right lobe graft usually provides an adequate liver volume to overcome the SFSS, but it is frequently associated with considerable morbidity to the donor.^[7,8]^ The left lobe graft is usually utilized in selected small-size recipients with comparable outcomes to the right lobe graft.^[9]^ Moreover, the feasibility of right posterior section as an alternative option to overcome the disproportionate size of the lobes of the liver has also been utilized with promising results.^[10,11]^ Until recently however, Suh et al ^[12]^ first described their experienced in utilizing the anterior section graft when both left lobe graft and right posterior lobe graft of the donor were insufficient to satisfy the metabolic demand of the recipient, and that the procurement of the right lobe graft could compromise safety of the donor. Thus, faced with this unavoidable scenario, we believe that an innovative selection of the right anterior section graft to balance the risk to the donor and recipient could represent a possible approach. Herein, we describe our first successful LDTL using right anterior section graft in a patient with end-stage liver disease secondary to severe alcoholic liver cirrhosis.

## 2 Case report

A 56-year-old man, a heavy alcoholic beverage drinker for 20 years, was diagnosed with alcoholic liver cirrhosis for almost 3 years. He was previously admitted twice on May 2016 and November 2016, for bleeding esophageal varices. On March 23, 2018, he presented a hepatic encephalopathy coma with massive ascites and was treated accordingly. Thus, an LDTL was offered to the patient as the best treatment option available.

Preoperatively, his body weight was 78.8 kg with a body mass index (BMI) of 27.6. The Child–Turcotte–Pugh score was 11 (class C), and the Model for End-stage Liver Disease (MELD) score was 21.62.

**Figure 1:**
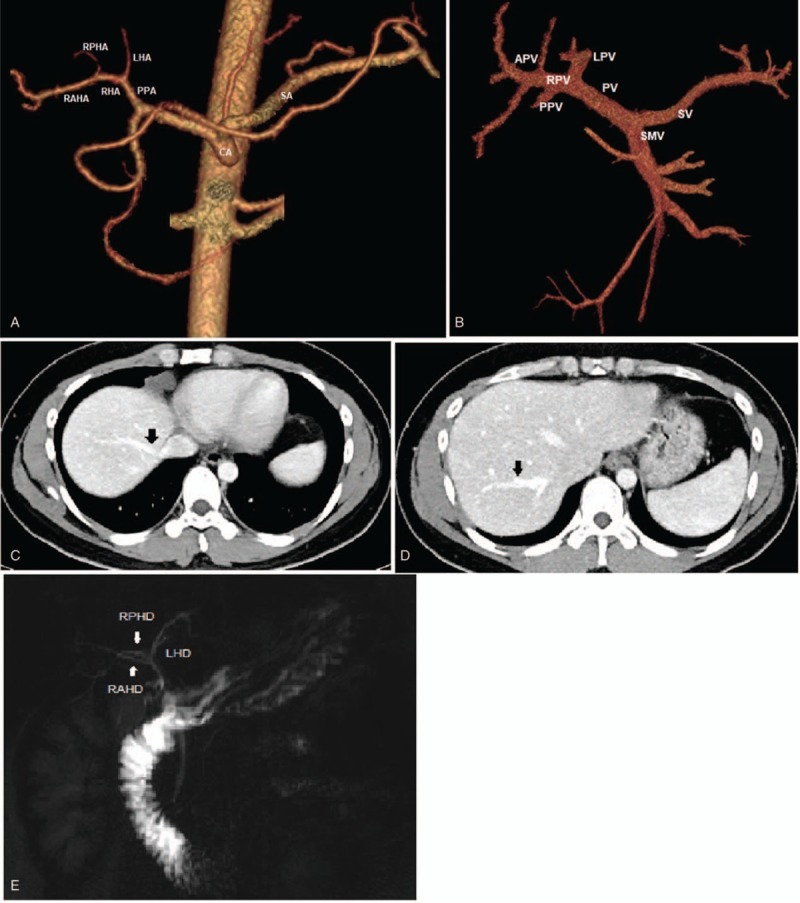
Preoperative imaging studies. Computed tomography (CT) scan three-dimensional reconstruction showing a normal variant of the hepatic artery (A) and portal vein (B). The tributaries of the right hepatic vein (RHV) (black arrows) draining segment VII (C) and segment 5 (D). (E) The trifurcation of the biliary tree was noted on MRCP.

The patient's 26-year-old son was found to be the only donor-compatible candidate for the LDTL. Preoperative evaluation included multidetector computed tomography (CT) scan, magnetic resonance imaging (MRI) including magnetic resonance cholangiopancreatography, and liver fibrosis scan. The segmental branches of the portal vein and hepatic artery were normal (Fig. 1A, B). In addition, large tributaries of the RHV from segments V to VIII were documented (Fig. 1C, D) and a trifurcation of the hilar hepatic duct was identified (Fig. 1E). In the CT volumetric study, the total liver volume was 1260 cm^3^. The right lobe had a total volume of 960 cm^3^, which occupied 76.2% of the total liver volume. The estimated residual liver volume was 23.8% and the graft-to-recipient weight ratio (GWRW) was 1.21%. The liver fibrosis scan was essentially normal. The right anterior section was considered the graft of choice with an estimated liver volume of 655 mL, an estimated GWRW of 0.83%, and adequate remnant volume of 605 mL. Table 1 summarizes the volumetric assessment of the liver volume of the donor. Patients provided informed consent, and the institutional review board approved the study protocol with an approval number 4-2018-1168.

**Table 1:**
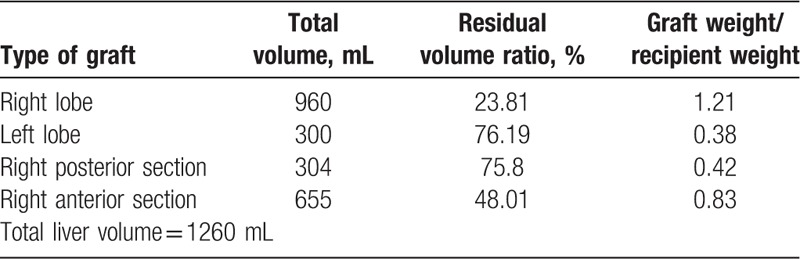
Preoperative multidetector CT volumetric assessment of the liver.

### 2.1 Donor anterior sectionectomy

**Figure 2:**
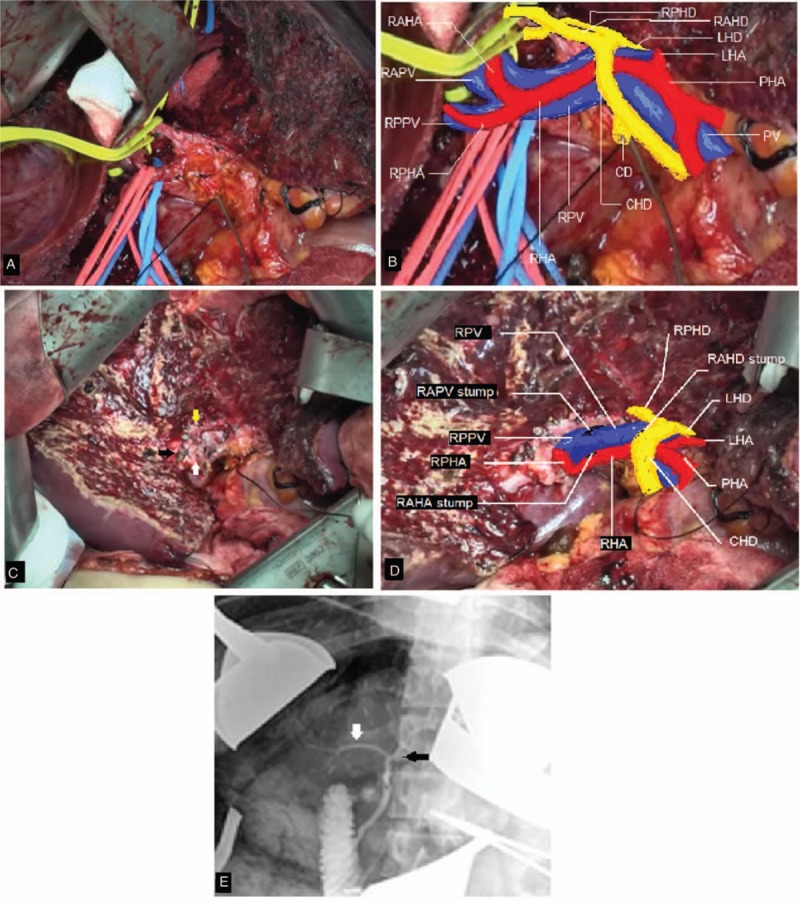
Intraoperative findings during right anterior sectionectomy. (A, B) Hilar structures were meticulously isolated and the exact locations of the right anterior hepatic duct (RAHD) and the right posterior hepatic duct (RPHD) were identified intraoperatively by probing method before transection of these structures. (C, D) The RPPV, right posterior hepatic artery (RPHA), and the RPHD were well preserved. (E) Completion cholangiogram showing an intact RPHD (white arrow) and LHD (black arrow).

A right subcostal incision with extension to the upper midline was made to access the abdomen. The ligamentum teres, falciform, coronary, and right triangular ligament were divided. Cholecystectomy was then performed. Hilar dissection was initiated posterolaterally to identify and isolate the right hepatic artery (RHA), right portal vein (RPV), and right hepatic duct (RHD). Likewise, the anterior and posterior branches of the RHA and RPV were identified and isolated. The right anterior portal vein (RAPV) and right anterior hepatic artery (RAHA) were temporarily clamped to make a demarcation line on the liver for parenchymal dissection. Intraoperative ultrasonography was also used to precisely confirm the location of the RHV and middle hepatic vein (MHV). The liver transection was performed using the Cavitron Ultrasonic Surgical Aspirator (CUSA). The RHV tributaries from segment V and VIII were carefully ligated and divided for recipient graft reconstruction. The intrahepatic course of the MHV was followed up to its confluence with the left hepatic vein. The right anterior hepatic duct (RAHD) was isolated using the Glissonian pedicle approach, and was sharply transected leaving behind approximately a 3-mm hepatic duct stump for subsequent duct-to-duct reconstruction (Fig. 2A, B). The RAHA and RAPV were ligated and transected. The MHV was immediately secured using an endovascular staple. The intraoperative image of the liver after anterior sectionectomy is shown in Fig. 2C, D. An intraoperative cholangiography was utilized to visualize the biliary tree, particularly to define the structural integrity of the right posterior hepatic duct (RPHD) (Fig. 2E).

### 2.2 Back table graft reconstruction

**Figure 3:**
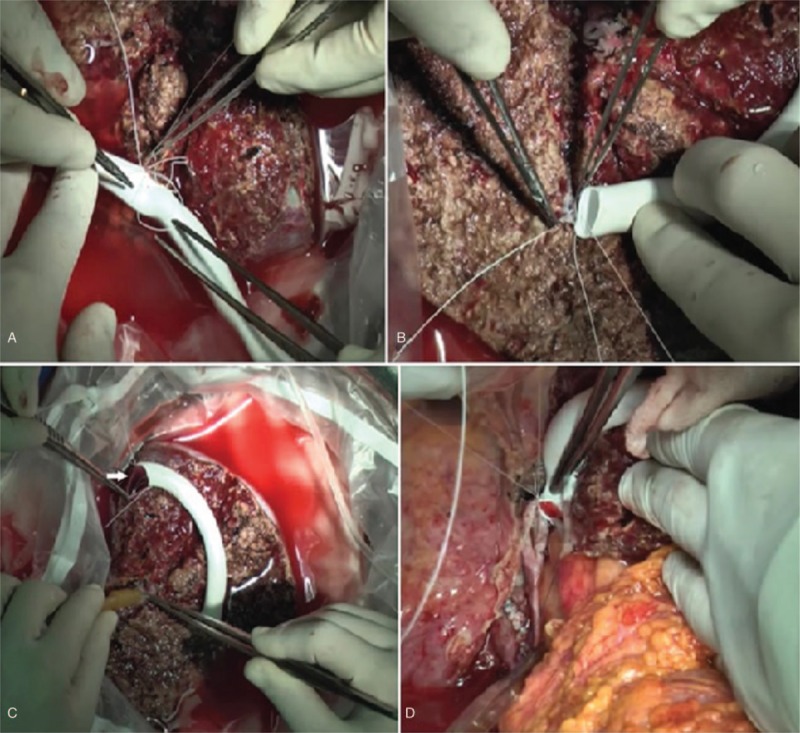
Back table graft reconstruction. The tributaries of the right hepatic vein (RHV) draining segment V (A) and segment VIII (B) were reconstructed using an interposition graft. (C) The interposition graft was anastomosed to the middle hepatic vein (MHV) (white arrow). (D) Venous outflow reconstruction. Venotomy was created to enlarge the orifice of the MHV (black arrow).

The right anterior section graft was flushed with a histidine–tryptophan–ketoglutarate solution. A vascular interposition graft was used to reconstruct the tributaries of the RHV from segments V and VIII (Fig. 3A, B). The proximal end of the interposition graft was conjoined with the MHV to form a common channel for venous outflow reconstruction (Fig. 3C).

### 2.3 Recipient surgery

A total hepatectomy with venacaval preservation was performed. A meticulous high hilar dissection was suitably performed. The stump of the MHV was made available for graft reconstruction. A venotomy was created from the orifice of the MHV to the inferior vena cava to enlarge the opening to ensure adequate venous outflow.

**Figure 4:**
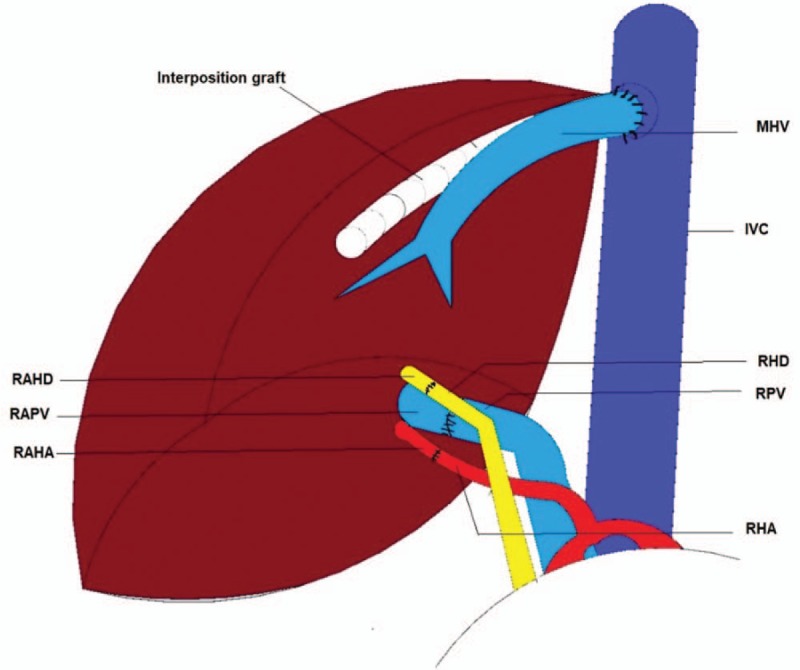
Schematic presentation of the transplanted anterior section graft.

Next, we performed hepatic venous outflow reconstruction between the enlarged orifice of the MHV stump and the vena cava of the recipient and the conjoined MHV and interposition graft of the donor (Fig. 3D). The RPV of the recipient was anastomosed to the RAPV of the donor. Microanastomosis between the donor's RAHA and the recipient's RHA was performed. A successful flow was confirmed by an intraoperative duplex ultrasonography. Finally, we anastomosed the recipient's RAHD to the donor's RHD using a duct-to-duct method. Figure 4 illustrates the transplanted anterior section graft of the recipient.

The total duration of surgery was 378 minutes with a total blood loss of 4100 mL. A volume of 1400 mL of ascites was noted intraoperatively with a grossly cirrhotic liver weighing 981 g. The right anterior section graft had an actual weight of 612 g, and the actual GRWR was 0.78. There was a 7% overestimation of the preoperative GWRW (0.83) compared with the actual GWRW (0.78) value.

### 2.4 Postoperative recovery

**Figure 5:**
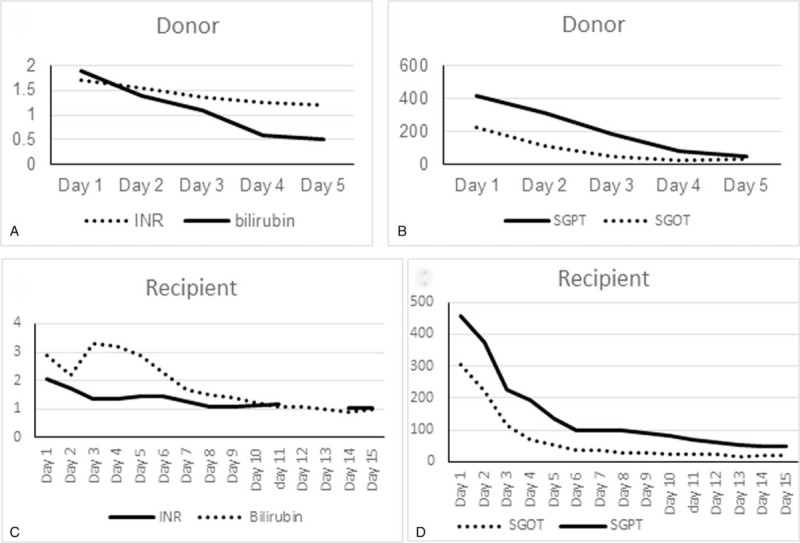
Functional liver recovery. (A) The INR and total bilirubin of the donor had returned to normal levels after the 5th postoperative day. (B) Initial rise of liver enzymes (alanine aminotransferase [SGPT] and aspartate aminotransferase [SGOT]) after surgery and return to normal at the 7th postoperative day. The INR activity and bilirubin level (C), as well as SGPT and SGOT (D) subsequently achieved normal levels after 15 days.

**Figure 6:**
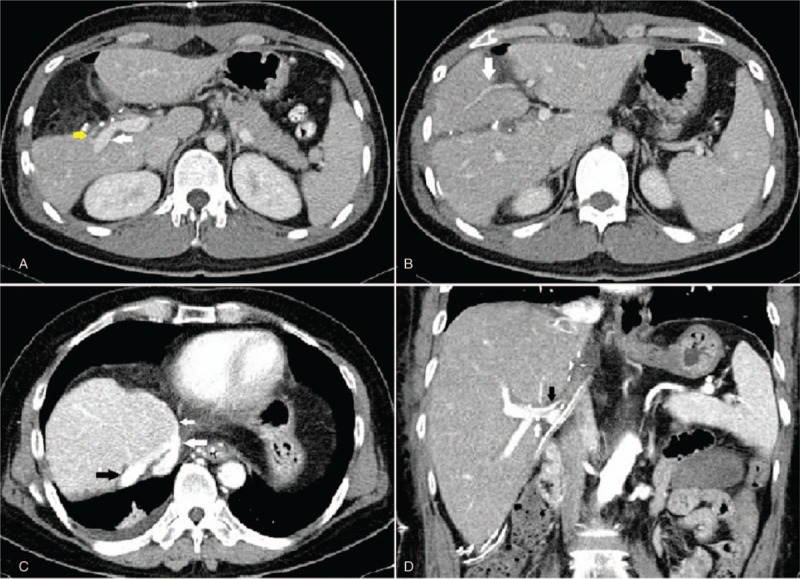
Postoperative imaging studies. (A) The donor's RPPV (white arrow) and the right posterior hepatic artery (RPHA) (yellow arrows) were intact. (B) A segmental branch of the LPV (white arrow). (C) The anastomotic site of the common channel formed by the graft (black arrow) and the middle hepatic vein (MHV) (white arrow) was intact. (D) The right anterior portal vein (RAPV) (white arrow) and the right anterior hepatic artery (RAHA) (black arrow) were also normal.

The donor was discharged on the 7th day after surgery. There was no report of postoperative complications such as bleeding, bile leakage, or hepatic insufficiency. Although there was a small focal liver congestion on segment IV observed on the CT scan on the 7th postoperative day. Liver functions were within normal limits. Figure 5A, B shows the liver function recovery following right anterior sectionectomy. Notably, the postoperative CT scan showed no signs of biliary or vascular abnormalities (Fig. 6A, B).

Most importantly, the recipient had an unremarkable hospital stay. Liver function returned to normal levels at 2 weeks posttransplantation (Fig. 5C, D). There were no signs of SFSS such as ascites, coagulopathy, or renal failure. A serial duplex ultrasonography of the liver showed normal biliary and vascular findings. Moreover, CT scan findings revealed no remarkable complications on the 14th postoperative day (Fig. 6C, D). The patient was discharged after the 15th hospital day.

## 3 Discussion

An accurate preoperative estimate of the liver volume is an integral part in the selection of appropriate graft to optimize the patient's outcome following LDTL. As part of our protocol, we obtained a multidetector CT volumetric study to accurately estimate the graft volume for liver transplantation. A GWRW of >0.8% and residual volume of >35% are the minimum criteria for LDTL at our center. Thus, in the case presented, the preoperative evaluation had identified that the donor right anterior section was the only graft that satisfied the required criteria. The right lobe graft had a GWRW of 1.21%, but had a residual volume of 23.81% that could significantly increase the risk of hepatic insufficiency postoperatively of the donor.^[13–15]^ Moreover, the right posterior graft and the left lobe graft had a GWRW of <0.8% which could otherwise increase the risk of SFSS after liver transplantation.^[16]^

Nevertheless, we had successfully performed the living-donor right anterior section graft with a GWRW of 0.83% and a residual liver volume ratio 48.01%. The graft size was sufficient to sustain the metabolic demands of the recipient postoperatively.^[17,18]^ Also, the residual liver volume was adequate to ensure the safety of the donor.^[15]^ As reported by Leelaudomlipi et al,^[19]^ the right anterior segment has an average volume ratio of 37%, whereas volumes of the left lateral section, left medial section, caudate lobe, and right posterior section were 17%, 14%, 2%, and 30%, respectively. Thus, the right anterior section could be a promising alternative option in selected patients with a disproportionate distribution of liver mass.

Furthermore, in the performance of graft reconstruction, an interposition graft was utilized to drain the large (>5 mm diameter) tributaries of the RHV of segments V and VIII. The venous outflow of segments V and VIII were maximized by the MHV and the interposition graft, preventing the occurrence of liver congestion,^[20–22]^ which could possibly cause small-for-size syndrome.^[23]^ As a result, there were no signs of liver congestion based on the CT scan findings following the liver transplantation.

Moreover, preoperative evaluation of the biliary and vascular anatomy is of equal importance for the determination of graft volume. In particular, it should be emphasized that the right anterior section has a more complex anatomy,^[24,25]^ thus, its feasibility should be restricted to certain favorable anatomical variations. As described by Hwang et al,^[26]^ intrahepatic second-branches of the RHA, RPV, and RHD are contraindications for procurement of right posterior sector graft due to the complexity of the reconstruction. Conversely, Sugawara et al^[27]^ reported that anatomical variations can be carefully managed, and that there are no exclusion criteria for the procurement of right posterior graft. The same is true for right anterior section graft procurement. However, as this was our first experience of right anterior sector graft in an LDTL, we believe that anatomical variations should be restrictions to right anterior sectionectomy to ensure the patient's safety. Special anatomical variants, such as an independent posterior branch of the portal vein, hepatic artery, or bile duct would be more suitable when using the anterior section. Moreover, particular attention to the arterial supply to segment IV should be emphasized because of the considerable incidence of segment IV hepatic artery arising from the RHA.^[28]^ Nevertheless, our case was shown to have an independent posterior branch of the right hepatic duct and segment IV hepatic artery from the left hepatic artery. This anatomical variant is favorable for right anterior sectionectomy.

Another important issue of concern in the procurement of the anterior sector graft is the hepatic vein drainage of segment IV. Inevitable segment IV congestion will occur if the dominant hepatic vein drainage stems from the MHV.^[29–31]^ However, we carefully evaluated the venous outflow in segment IV in this case and it was found to be left hepatic vein dominant. As a result, only focal segment IV congestion was noted postoperatively.

In summary, this case shows that procurement of right anterior section graft is technically feasible in a properly selected patient. However, it should be emphasized that only volumetric analysis when using the anterior section should be avoided and proper preoperative evaluation of the biliary and vascular anatomy should always be considered. More importantly, the procedure should be performed in high volume liver centers with considerable experience in LDTL. This approach, however, should be reevaluated with regard to safety and feasibility based on a large number of patients with long-term follow-up data.

## Author contributions

**Conceptualization:** Jonathan Geograpo Navarro, Gi Hong Choi.

**Data curation:** Jonathan Geograpo Navarro, Gi Hong Choi, Myoung Soo Kim, Yoon Bin Jung, Jae Geun Lee.

**Methodology:** Jonathan Geograpo Navarro.

**Project administration:** Gi Hong Choi.

**Supervision:** Gi Hong Choi, Myoung Soo Kim.

**Validation:** Gi Hong Choi, Myoung Soo Kim, Yoon Bin Jung, Jae Geun Lee.

**Visualization:** Gi Hong Choi, Jae Geun Lee.

**Writing – original draft:** Jonathan Geograpo Navarro.

**Writing – review and editing:** Jonathan Geograpo Navarro, Gi Hong Choi, Myoung Soo Kim, Yoon Bin Jung, Jae Geun Lee.

Jonathan Geograpo Navarro orcid: 0000-0001-5435-2333.
